# Hyperpigmentation and chikungunya fever*

**DOI:** 10.1590/abd1806-4841.20165805

**Published:** 2016

**Authors:** Ankita Srivastava

**Affiliations:** 1 RUHS College of Medical sciences – Jaipur, India

Dear Sir,

Chikungunya fever (CF) is an acute febrile illness presenting with symptoms like intense asthenia, arthralgia, myalgia and headache and is caused by chikungunya virus (CV).^1,2^ Apart from fever, joint pain and other constitutional symptoms, various mucocutaneous changes also occur. Of these, maculopapular rash is common in several viral illnesses, therefore, not useful in suspecting CF. On the other hand, hyperpigmentation is a unique feature noted in CF. Knowledge of this pigmentary change is essential, both amongst the physicians and dermatologists, since it can act as an indicator for an undetected outbreak of CF; especially in a health set up where it is not possible to screen every case of viral fever for CF.

Hyperpigmentation associated with CF is macular and most commonly affects nose and cheeks. ^3,4^ It may develop soon after the rash has resolved, and has an acute onset. ^4^ Usually pigmentary changes develop after two weeks or more after the rash; by the time fever has subsided; hence it may be termed as post chikungunya pigmentation (PCP). This is the time when patient is most likely to visit a dermatologist. PCP may occur in the form of discrete macules, freckle-like, diffuse, flagellate, Addisonian type of palmar pigmentation, periorbital melanosis and pigmentation of pre-existing acne lesions. ^3,4^ Involvement of centrofacial face (nose and cheeks) mimics melasma and in a busy outpatient department (OPD), is likely to be missed especially if proper history is not taken. ^3^ Patients with PCP give a history of high grade fever 2-4 weeks before onset, acute onset of hypermelanosis and many times, persistent asthenia and joint pain even after defervescence of fever. All these points should alert the dermatologist to think of CF. Interestingly, these patients do not have any preceding erythema or eruption over the affected areas during the acute febrile phase. ^5^

The "chik sign": ^3^

**Figure 1:**
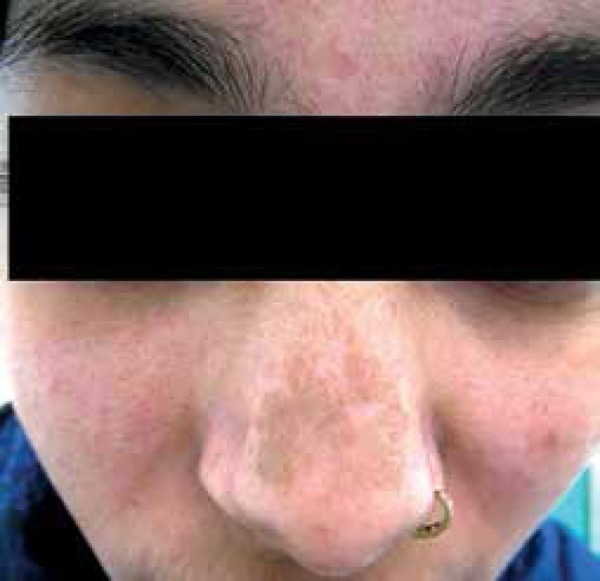
Pigmentation over nose – the chik sign

Striking pigmentation over nose is seen in several patients of CF, which has been termed as the "chik sign" by Riyaz et al. (Figure 1). Since this pigmentation may persist for some after the acute illness, it may be helpful in making a retrospective diagnosis of CF too. ^3^

Therefore, the learning point for the dermatologist is to rule out history of a febrile illness in patients with melasma-like pigmentation, especially in those who present with a short duration or acute onset. At the same time, physicians should also be alert to screen patients serologically for CF, especially those who developed hyperpigmentation in association with febrile episodes. Knowledge of this cutaneous feature is extremely useful in a resource-poor setting to detect an unrecognized outbreak of CF; where most cases of any viral fever are managed conservatively. Timely recognition of such outbreak is of utmost importance from the public health point of view; so that adequate vector control measures are adopted to prevent further spread of the disease.
